# Long‐term efficacy of rituximab versus intravenous cyclophosphamide for severe ANCA‐associated vasculitis in multicenter REVEAL cohort study

**DOI:** 10.1111/joim.70024

**Published:** 2025-09-22

**Authors:** Shogo Matsuda, Takuya Kotani, Daisuke Nishioka, Ayana Okazaki, Yuichi Masuda, Tomoki Taniguchi, Mikihito Shoji, Tsuneyasu Yoshida, Ryosuke Hiwa, Mayu Shiomi, Ryu Watanabe, Muneyuki Hatta, Naoko Ito, Yohei Fujiki, Hirofumi Miyake, Wataru Yamamoto, Motomu Hashimoto, Tohru Takeuchi

**Affiliations:** ^1^ Department of Internal Medicine IV, Division of Rheumatology Osaka Medical and Pharmaceutical University Osaka Japan; ^2^ Department of Medical Statistics, Research & Development Center Osaka Medical and Pharmaceutical University Osaka Japan; ^3^ Department of Internal Medicine IV, Division of Neurology Osaka Medical and Pharmaceutical University Osaka Japan; ^4^ Department of Rheumatology and Clinical Immunology, Graduate School of Medicine Kyoto University Kyoto Japan; ^5^ Department of Clinical Immunology Osaka Metropolitan University Osaka Japan; ^6^ Division of Clinical Immunology and Rheumatology Yodogawa Christian Hospital Osaka Japan; ^7^ Department of General Internal Medicine Tenri Hospital Nara Japan; ^8^ Department of Health Information Management Kurashiki Sweet Hospital Kurashiki Japan

**Keywords:** ANCA‐associated vasculitis, cyclophosphamide, IIPTW, prognosis, rituximab

## Abstract

**Background:**

The optimal remission induction therapy for severe antineutrophil cytoplasmic antibody‐associated vasculitis (AAV) remains unclear. This study evaluated the effectiveness of rituximab (RTX) as a remission induction therapy for severe AAV compared with intravenous cyclophosphamide (IVCY).

**Methods:**

Patients with microscopic polyangiitis (MPA) and granulomatosis with polyangiitis (GPA) treated with systemic glucocorticoids (GCs) and IVCY or RTX as initial remission induction therapy in multicenter REVEAL study between 1991 and 2024 were enrolled. We compared efficacy and safety outcomes between the two groups. Effectiveness was evaluated using all‐cause mortality, GC‐remission rate, relapse rate, and end‐stage renal disease (ESRD) progression rate. Safety was evaluated based on complications from severe infections. Inverse probability of treatment weighting (IPTW) was applied for selection bias.

**Results:**

Of 555 patients with AAV, 178 with severe MPA or GPA were identified (IVCY group: *N* = 133, RTX group: *N* = 45). After adjustment by IPTW, no significant differences in baseline clinical characteristics were observed between them. The 10‐year survival rate and GC‐remission rate at 6 months were significantly higher in the RTX group (*p* = 0.04, *p* = 0.017, respectively). ESRD progression and relapse rates were comparable between two groups. Regarding safety, 15.2% of patients in the IVCY group died due to severe infections, whereas none did in the RTX group (*p* = 0.007).

**Conclusions:**

RTX demonstrated superior efficacy in improving survival and achieving GC remission, with fewer infection‐related deaths compared to IVCY in patients with severe AAV. These findings reveal the efficacy and safety of RTX as a remission induction therapy in real‐world Japanese clinical practice.

## Graphical abstract

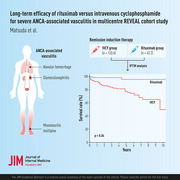

## Introduction

Anti‐neutrophil cytoplasmic antibody (ANCA)‐associated vasculitis (AAV) is a systemic necrotizing small‐vessel vasculitis [1]. Microscopic polyangiitis (MPA), granulomatosis with polyangiitis (GPA), and eosinophilic granulomatosis with polyangiitis (EGPA) are the three major clinicopathological subtypes of AAV. Patients with AAV and life‐threatening organ lesions, including rapidly progressive glomerulonephritis and alveolar hemorrhage, have a poor prognosis [2, 3]. Systemic glucocorticoids (GCs) in combination with intravenous cyclophosphamide (IVCY) or rituximab (RTX) are recommended as remission‐induction therapy for patients with severe AAV [4].

Two randomized clinical trials, the RAVE and RITUXVAS trials, showed the non‐inferiority of RTX to IVCY in terms of the remission rate and incidence of side effects at 6 and 12 months after induction therapy [5, 6]. However, these RCTs use inclusion and exclusion criteria, which may limit the generalizability of these results to real‐world clinical practice [7]. After these RCTs, several observational studies and post hoc analyses have examined the differences in efficacy and safety between RTX and IVCY treatments in patients with AAV [8, 9, 10, 11, 12, 13]. However, owing to variations in patient backgrounds, such as racial differences, disease distribution, and follow‐up periods, the efficacy and safety of both groups differ across studies. Whether RTX is more effective than IVCY in patients with severe AAV in a long‐term real‐world setting remains unknown.

Multicenter studies using real‐world data increase generalizability; however, selection bias due to uncontrolled differences remains a concern. Inverse probability of treatment weighting (IPTW) is a popular method for reducing selection bias by adjusting for potential confounding factors [14]. In the present study, we adjusted the patient backgrounds of the RTX and IVCY groups using IPTW analysis and investigated the effectiveness and safety of RTX as a remission induction therapy for severe AAV compared with IVCY using a Japanese multicenter registry of vasculitis patients to establish real‐world evidence (REVEAL) cohort database.

## Methods

### Patients

This multicenter, observational, retrospective study was conducted using the REVEAL cohort to compare the effectiveness and safety of RTX and IVCY. The REVEAL cohort is an observational multicenter registry involving patients with AAV, including those with MPA, GPA, and EGPA, in Japan [15, 16, 17]. We included data from five participating institutes (Osaka Medical and Pharmaceutical University, Kyoto University, Osaka Metropolitan University, Yodogawa Christian Hospital, and Tenri Hospital). Between May 1991 and July 2024, 555 patients with AAV were enrolled. The patients were diagnosed with MPA/GPA/EGPA based on the Chapel Hill Consensus definition [18]. Severe AAV was defined as the presence of one or more major items on the Birmingham Vasculitis Activity Score (BVAS) version 3, or severe manifestations requiring treatment with IVCY or RTX to induce remission induction therapy, as based on previous studies [6, 19]. The typical severe manifestations were glomerulonephritis, alveolar hemorrhage, vasculitic neuropathy, gastrointestinal involvements, and cardiac involvement, as referred previously. [20, 21] At enrollment, clinical data for remission induction therapy and outcomes, which were recorded at admission, were retrospectively collected from the electronic database by a reference clinician at each center. This study was conducted in accordance with the Declaration of Helsinki and its amendments. Moreover, it was approved by the Osaka Medical and Pharmaceutical University and the Faculty of Medicine Ethics Committee (approval no. 1529) and by the participating centers, including Kyoto University (approval no. R1540), Osaka Metropolitan University (approval no. 2023‐027), Yodogawa Christian Hospital (approval no. 2023‐070), and Tenri Hospital (approval no. C23‐11). The Ethics Committees of Kyoto University and Osaka Metropolitan University waived the requirement for informed consent because of the anonymous nature of the data. Written informed consent was obtained from each patient from the other institutions.

### Clinical findings, laboratory parameters, and evaluation of disease severity on admission

In the REVEAL cohort database, the following data were collected from the medical records: patient demographic characteristics, including age at admission and sex; peripheral laboratory data, including white blood cell (WBC) counts, C‐reactive protein (CRP), serum creatinine (Cr), myeloperoxidase (MPO)‐ANCA, and proteinase 3 (PR3)‐ANCA; systemic organ involvement defined by the BVAS, version 3 [22]; and the treatment details. Total BVAS scores were collected to evaluate systemic disease activity. According to the European Vasculitis Study Group (EUVAS) categorization system, disease severity states, including localized, early systemic, systemic, and severe, were collected [23]. Interstitial lung disease (ILD) was evaluated using chest high‐resolution computed tomography scans obtained by pulmonary radiologists at each institution.

## Outcome

The primary outcome of this study was all‐cause mortality. To evaluate outcomes, follow‐up survival data were collected retrospectively from the REVEAL cohort, and all‐cause mortality was examined. Secondary outcomes were GC‐remission, end‐stage renal disease (ESRD) rates, and relapse rates. Patients were considered to have GC remission when they met two criteria: (1) a BVAS of 0 on two occasions at least 1 month apart, and (2) a daily prednisolone (PDN) dosage of ≤10 mg, as defined by Sada et al. [24]. Relapse was defined as the recurrence of vasculitis requiring a treatment change, increasing the dose of GCs, and/or adding immunosuppressants due to the exacerbation of symptoms and clinical data, as previously described [25]. ESRD in patients with AAV was defined as meeting either of the following two criteria: (1) an estimated glomerular filtration rate (eGFR) of less than 15 mL/min/1.73 m^2^ with a requirement for permanent renal replacement therapy or (2) dependence on hemodialysis for more than 3 months from the time of AAV diagnosis [26]. Infection‐related deaths and hospitalizations were examined in this study.

### Statistical analysis

Data are presented as medians and interquartile ranges. Fisher's exact test was used when appropriate, and the Mann–Whitney *U* test was used to compare the median values. Statistical significance was set at *p* < 0.05. Stabilized IPTW based on the propensity score was performed to control for potential confounding factors between the IVCY and RTX groups. A logistic regression model was used to calculate the propensity scores. The model was based on the following potential confounding variables: age, sex, proportion of MPA and ILD, total BVAS scores, serum WBC count, serum CRP levels, serum Cr levels, proportion of organ involvement, EUVAS‐defined disease activity, initial PDN dose, and proportion of methylprednisolone (MPDN) pulse therapy. These confounding variables are complete data with no missing values in this study. Standardized differences were calculated to compare the confounders between the IVCY and RTX groups. An absolute standardized difference of <0.10 denoted a negligible difference between the two groups, as previously described [27]. Categorical variables were compared using the chi‐square test and continuous variables using the Mann–Whitney *U* test before IPTW, and weights were considered in the IPTW analysis. The Kaplan–Meier method was used to assess survival, relapse‐free survival, and ESRD‐free survival, and the log‐rank test was used to evaluate the significance of differences between groups. Survival, relapse‐free survival, and ESRD‐free survival times were calculated as the period between the date of remission induction therapy at each institution and the latest hospital visit, date of censoring, time of death, time of relapse, or time of ESRD. We also estimated the hazard ratios (HRs) of the patient outcomes using a Cox regression model. Fine–Gray regression analysis was performed to account for the competing risk of death. The eGFR slope was calculated using a linear mixed‐effects model, as described in the previous paper [28]. The 1‐year eGFR slope was calculated using measurements taken at baseline (year 0) and at a follow‐up visit 1 year (±3 months) later, as previously reported [29]. All available eGFR data from this period were used to calculate the eGFR slope. Data were analyzed using JMP (version 17.0; SAS Institute Inc.), GraphPad Prism (version 8.0; GraphPad Software), and Easy R software (Saitama Medical Center, Jichi Medical University) [30].

## Results

### Participant selection

**Fig. 1:**
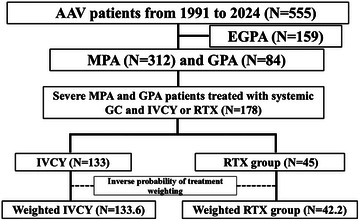
**Flowchart of this study**. AAV, anti‐neutrophil cytoplasmic antibody‐associated vasculitis; EGPA, eosinophilic granulomatosis with polyangiitis; GC, glucocorticoid; GPA, granulomatosis with polyangiitis; IVCY, intravenous cyclophosphamide; MPA, microscopic polyangiitis; RTX, rituximab.

In the REVEAL cohort, 555 patients with AAV were registered from May 1991 to September 2024. A total of 312, 84, and 159 patients were diagnosed with MPA, GPA, and EGPA, respectively. We selected patients diagnosed with MPA and GPA who were treated with systemic GC therapy and immunosuppressants (IVCY or RTX). The patients with EGPA were excluded from the analysis as the clinical phenotype of EGPA is markedly different from that of MPA and GPA [31]. Therefore, 178 patients with severe AAV were included in this study (IVCY group: *N* = 133, RTX group: *N* = 45) (Fig. 1). The clinical characteristics of these patients are summarized in Table S1. The median age of the patients was 74 (67–79) years, and 56.2% were female. The proportions of MPA and GPA were 80.9% and 19.1%, respectively. Of these, 87% and 11.2% were positive for MPO‐ANCA and PR3‐ANCA, respectively. The medians of total BVAS score, initial WBC count, CRP level, and serum Cr levels were 17 (12–20), 11,325 (8083–14,335)/mm^3^, 8.6 (2.4–13.8) mg/dL, and 1.13 (0.71–2.08) mg/dL, respectively. The proportions of patients with localized, early systemic, systemic, and severe disease were 1.7%, 19.7%, 62.4%, and 16.3%, respectively. As remission induction therapy, the median initial PDN dose was 1.0 (0.96–1.0) mg/kg, and 39.3% were treated with MPDN pulse therapy. Maintenance therapy was shown in Table S2. Azathioprine (AZP) was used in 108 cases (60.7%), methotrexate (MTX) in 10 (5.6%), mycophenolate mofetil (MMF) in 10 (5.6%), tacrolimus (TAC) in 7 (3.9%), mizoribine (MZB) in 3 (1.7%), and cyclosporine (CyA) in 4 (2.2%). The median duration of AZP, MTX, MMF, TAC, MZB, and CyA was 2.9, 1.0, 3.7, 1.7, 0.4, 3.1 years, respectively. RTX was used for maintenance therapy in 20 cases (11.2%).

### Patient characteristics before and after IPTW

Patient characteristics before and after IPTW analysis are shown in Table 1.

**Table 1:** Baseline characteristics of IVCY group and RTX group in patients with MPA and GPA before and after IPTW.

	Before IPTW				After IPTW			
Variables	IVCY (*N* = 133)	RTX (*N* = 45)	*p* value	ASD	IVCY (*N* = 133.6)	RTX (*N* = 42.2)	*p* value	ASD
Age, years	73.0 (66.0–78.3)	76.9 (71.0–80.0)	0.03^*^	0.21	73.0 (66.0–78.1)	76.0 (64.8–79.0)	0.38	0.04
Female, *n* (%)	74 (55.6)	26 (57.8)	0.94	0.04	73.9 (55.3)	22.3 (52.7)	0.81	0.05
MPA, *n* (%)	112 (84.2)	32 (71.1)	0.09	0.32	105.6 (79.0)	32.4 (76.8)	0.79	0.05
ILD, *n* (%)	65 (48.9)	10 (22.2)	0.003^**^	0.58	56.6 (42.4)	18.5 (43.9)	0.89	0.03
BVAS at onset	16.0 (12.0–20.0)	18.0 (12.0–20.0)	0.98	0.003	16.0 (12.0–20.0)	18 (12.0–22.2)	0.39	0.19
**Laboratory findings**								
WBC, /mm^3^	10,350 (7900–14,000)	13,100 (9240–15,390)	0.08	0.24	10,457 (8062–14,300)	12,351 (8932–14,753)	0.22	0.17
CRP, mg/dL	8.05 (2.50–13.70)	10.1 (2.44–14.31)	0.44	0.18	7.98 (2.29–13.81)	9.65 (2.4–11.7)	0.66	0.09
Cr, mg/dL	1.08 (0.70–1.83)	1.26 (0.8–3.2)	0.038^*^	0.37	1.06 (0.68–1.92)	1.22 (0.8–2.1)	0.35	0.03
**Organ involvements**								
General, *n* (%)	94 (70.7)	26 (57.8)	0.16	0.27	87.3 (65.3)	27.3 (64.7)	0.95	0.01
Cutaneous, *n* (%)	14 (10.5)	6 (13.3)	0.81	0.09	14.9 (11.1)	4.6 (10.9)	0.97	0.007
Mucous membranes/eyes, *n* (%)	14 (10.5)	6 (13.3)	0.81	0.09	13.5 (10.1)	3.4 (8.0)	0.66	0.07
ENT, *n* (%)	30 (22.6)	16 (35.6)	0.13	0.29	36.4 (27.3)	13.4 (31.8)	0.63	0.09
Chest, *n* (%)	70 (52.6)	23 (51.1)	0.99	0.03	71.8 (53.8)	21.4 (50.8)	0.78	0.06
Cardiovascular, *n* (%)	6 (4.5)	1 (2.2)	0.81	0.13	5.3 (4.0)	1.9 (4.4)	0.92	0.02
Abdominal, *n* (%)	1 (0.8)	1 (2.2)	1	0.12	1.0 (0.8)	0.4 (0.8)	0.96	0.006
Renal, *n* (%)	101 (75.9)	33 (73.3)	0.88	0.06	96.8 (72.5)	31.4 (74.4)	0.83	0.04
Nervous system, *n* (%)	55 (41.4)	16 (35.6)	0.61	0.12	53.1 (39.8)	20.3 (48.1)	0.44	0.17
**EUVAS‐defined disease activity**								
Localized, *n* (%)	3 (2.3)	0 (0.0)	0.73	0.22	2.2 (1.7)	0 (0.0)	0.34	0.19
Early systemic, *n* (%)	22 (16.5)	13 (28.9)	0.11	0.3	28.7 (21.5)	10.1 (23.9)	0.77	0.06
Systemic, *n* (%)	89 (66.9)	22 (48.9)	0.048^*^	0.37	81.8 (61.2)	26.1 (61.9)	0.95	0.01
Severe, *n* (%)	19 (14.3)	10 (22.2)	0.31	0.21	20.9 (15.6)	6.0 (14.2)	0.81	0.04
**Treatments**								
Initial PDN dose, mg/kg	1.0 (0.98–1.00)	1.0 (0.93–1.00)	0.49	0.22	1.0 (0.86–1.00)	1.0 (0.96–1.00)	0.82	0.02
MPDN pulse, *n* (%)	48 (36.1)	22 (48.9)	0.18	0.26	50.3 (37.7)	17.1 (40.5)	0.79	0.06

*Note*: The laboratory markers are presented as the median (interquartile range). The *p* values were estimated using the chi‐squared test or the Mann–Whitney *U* test.

Abbreviations: ASD, absolute standardized difference; BVAS, Birmingham Vasculitis Activity Score; Cr, creatinine; CRP, C‐reactive protein; ENT, ear, nose, and throat; EUVAS, European Vasculitis Study Group; GPA, granulomatosis with polyangiitis; ILD, interstitial lung disease; IPTW, inverse probability of treatment weighting; IVCY, intravenous cyclophosphamide; MPA, microscopic polyangiitis; MPDN, methylprednisolone; PDN, prednisolone; RTX, rituximab; WBC, white blood cell.

* *p* < 0.05.

** *p* < 0.01.

**Fig. 2:**
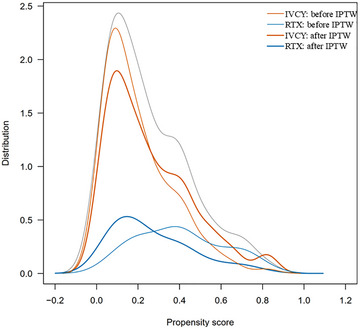
**Density curves depicting the before IPTW and after IPTW propensity score distribution in the IVCY and RTX groups**. IPTW, inverse probability of treatment weighting; IVCY, intravenous cyclophosphamide; RTX, rituximab.

Before IPTW, the median age and serum Cr levels were significantly higher in the RTX group (76.9 years, 1.26 mg/dL) compared to the IVCY group (73.0 years, 1.08 mg/dL) (*p* = 0.03, 0.038, respectively). Additionally, ILD prevalence and systemic involvement proportions were significantly lower in the RTX group (22.2%, 48.9%) than in the IVCY group (48.9%, 66.9%) (*p* = 0.003, 0.048, respectively). Several factors were unbalanced between the IVCY and RTX groups (absolute values of standardized difference >0.1), including age, proportion of MPA and ILD, inflammatory markers (WBC and CRP levels), serum creatinine, proportions of organ involvement (general; ear, nose, and throat; cardiovascular; abdominal; and nervous system), EUVAS‐defined disease severity, and treatment details. After IPTW, absolute values of standardized differences for all the variables except BVAS, WBC levels, proportions of the nervous system, and proportions of “localized” were ≦0.1. Density curves for the distribution of propensity scores before and after IPTW in the IVCY and RTX groups are shown in Fig. 2.

## Outcomes

### Primary outcome: mortality rate

**Fig. 3:**
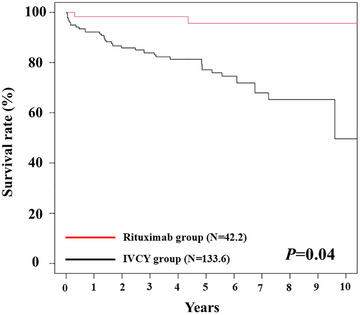
**Over survival rates in the IVCY and RTX groups after IPTW analysis**. IPTW, inverse probability of treatment weighting; IVCY, intravenous cyclophosphamide; RTX, rituximab.

After the IPTW analysis, we compared the mortality rates between the RTX and IVCY groups, which was the primary outcome. The IPTW‐weighted results showed that 41.7 patients (31.2%) died in the IVCY group, whereas 0.95 patients (2.3%) died in the RTX group. The causes of death are listed in Table S3. The Kaplan–Meier survival curves for the RTX and IVCY groups after IPTW analysis are shown in Fig. 3. The 10‐year survival rate was significantly higher in the RTX group than in the IVCY group (*p* = 0.04, log‐rank test). Cox logistic regression analyses after IPTW also showed that the RTX group had significantly higher survival rates than the IVCY group (HR: 0.10 [0.02–0.47], *p* = 0.004).

As patient attrition due to hospital transfers poses a competitive risk, the mortality rate was reanalyzed using a competing‐risk analysis model. Fine–Gray regression model also showed the RTX group had higher survival rates compared to IVCY group (HR: 0.03 [0.005–0.18], *p* = 0.0001).

### Secondary outcomes: GC‐remission, relapse, and ESRD rates

**Fig. 4:**
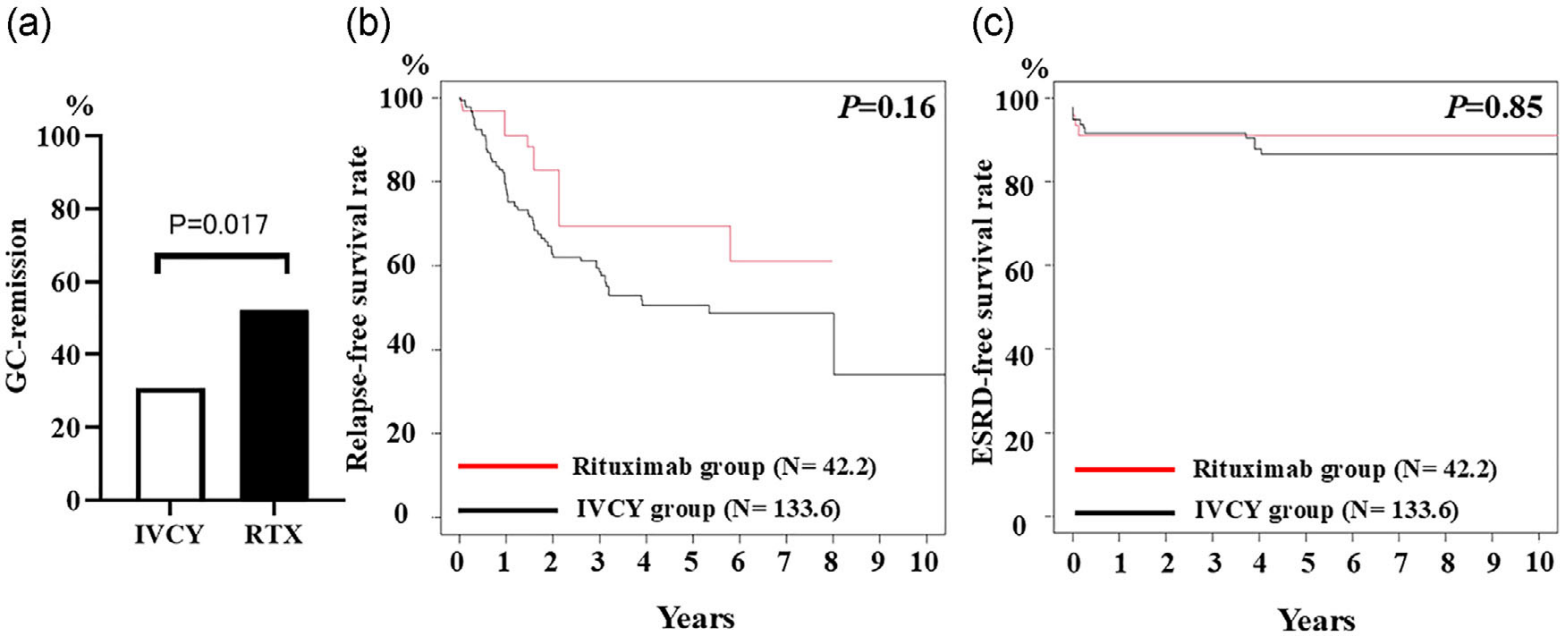
**GC‐remission rates, relapse rates, and ESRD rates in the IVCY and RTX groups after IPTW analysis**: (a) GC‐remission ratios at month 6, (b) relapse‐free survival rates, and (c) ESRD‐free survival rates in the IVCY and RTX groups after IPTW analysis. ESRD, end‐stage renal disease; GC, glucocorticoid; IPTW, inverse probability of treatment weighting; IVCY, intravenous cyclophosphamide; RTX, rituximab.

Secondary outcomes, including GC‐remission, relapse, and ESRD rates, were compared between the RTX and IVCY groups after IPTW analysis. The GC‐remission rate at 6 months was higher in the RTX group (52.1%) than in the IVCY group (30.9%) (*p* = 0.017) (Fig. 4a).

Regarding relapse, the IPTW‐weighted results showed that 59.9 patients (44.9%) in the IVCY group and 7.5 patients (17.8%) in the RTX group experienced relapse during follow‐up. At the time of relapse, 49.4% of the IVCY group and 49.0% of the RTX group received immunosuppressive therapy (IS) as maintenance therapy. The detail of IS in each group was shown in Fig. S1. Ten‐year relapse rates tended to be lower in the RTX group than in the IVCY group (*p* = 0.16), but the difference was not significant (Fig. 4b). In the IVCY group (*n* = 59.9), 49 patients (81.8%) were treated with an increased dose of PDN, and 31.4 patients (52.4%) were treated with additional IS. In the RTX group (*n* = 7.5), 6.2 patients (82.4%) received a PDN dose increase, and 1.3 patients (17.6%) were treated with additional IS.

Finally, we evaluated the ESRD progression rates in both groups. The IPTW‐weighted results showed that 13.8 patients (10.4%) and 2.9 patients (6.9%) in the IVCY and RTX groups progressed to ESRD, respectively. Ten‐year ESRD progression rates were comparable between the two groups (*p* = 0.85) (Fig. 4c).

### Safety

We examined the proportion of infection‐related mortality and hospitalizations in the IVCY and RTX groups after IPTW analysis. The IPTW‐weighted results showed that there were 20.3 deaths (15.2%) due to severe infection in the IVCY group, but none in the RTX group (*p* = 0.007). In the IVCY group, severe infections occurred at a median of 1.97 years (0.69–7.5 years) after induction therapy. Among severe infections in the IVCY group, 70.2% of deaths were due to respiratory infections, and 20.8% of deaths were due to sepsis. The causes of respiratory infections were bacterial pneumonia (78.3%), viral pneumonia (10.8%), and fungal pneumonia (10.9%).

The proportions of infection‐related hospitalizations were 16.1% and 30.7% in the RTX and IVCY groups, respectively, showing a higher proportion in the IVCY group; however, this difference was not statistically significant (*p* = 0.06). The details of infections that caused hospitalization for the RTX and IVCY groups were shown in Fig. S2. The median time from remission induction to infection‐related hospitalization was 0.31 years (0.19–3.77) for the RTX group and 2.35 years (1.17–5.07) for the IVCY group. The median hospitalization period was 0.43 months (0.43–1.78) for the RTX group and 0.9 months (0.47–1.63) for the IVCY group. There was no significant difference in the time from remission induction to infection‐related hospitalization or in the median hospitalization period between the RTX and IVCY groups (*p* = 0.11, 0.43).

### Serial changes of PDN dose and BVAS in the RTX and IVCY groups

**Fig. 5:**
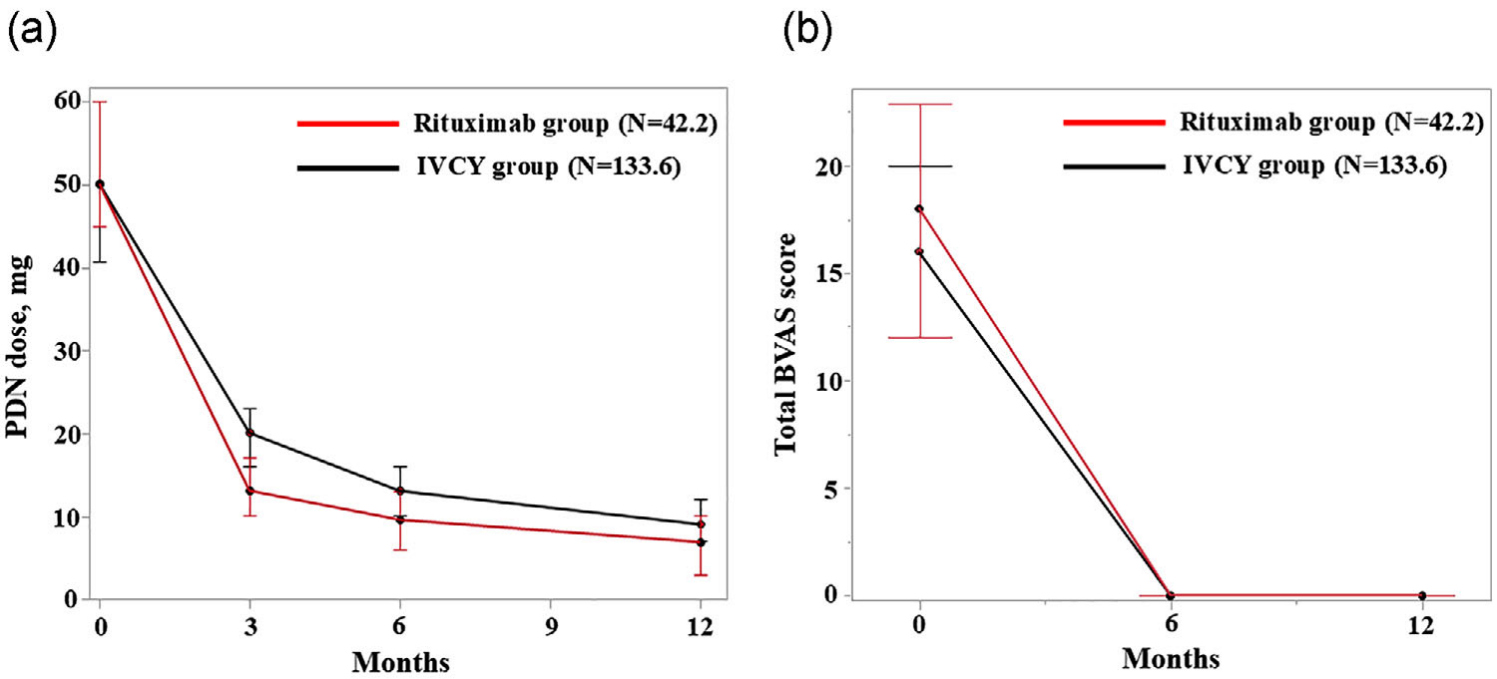
**Serial changes of PDN dose and BVAS in the IVCY and RTX groups after IPTW analysis**. (a) Serial changes of PDN dose at 0 months, 3 months, 6 months, and 12 months. (b) Serial changes of BVAS scores at 0 months, 6 months, and 12 months. Lower line = first quartile. Upper line = third quartile. BVAS, Birmingham Vasculitis Activity Score; IVCY, intravenous cyclophosphamide; PDN, prednisolone; RTX, rituximab.

In this study, the GC‐remission rate at 6 months was significantly lower in the RTX group than in the IVCY group. To examine the GC tapering speed, we collected time‐course PDN doses at 3, 6, and 12 months in the IVCY and RTX groups (Fig. 5). The median PDN dose at 3 months was 20 (16–23) mg/day in the IVCY group and 13 (10–17) mg/day in the RTX group. At 6 months, the median PDN dose was 13 (10–16) mg/day in the IVCY group and 9.5 (6–13) mg/day in the RTX group.

At 12 months, the median PDN dose was 9 (7–12) mg/day in the IVCY group and 6.8 (3–10) mg/day in the RTX group (Fig. 5a). The total BVAS scores at 6 and 12 months showed a significant decrease in both groups compared with the total BVAS score before treatment (Fig. 5b). These results suggest that in the RTX group, PDN could be tapered earlier than that in the IVCY group without exacerbating the disease.

### Comparison of 1‐year eGFR slope between the IVCY and RTX groups

We calculated the 1‐year eGFR slope and examined the difference between the IVCY and RTX groups. The median 1‐year eGFR slope estimated by a mixed‐effects model was −2.7 (−7.7 to 2.3) mL/min/1.73 m^2^ per year in RTX group and −1.3 (−4.1 to 1.6) mL/min/1.73 m^2^ per year in IVCY group. There was no significant difference in eGFR slope between the IVCY and RTX groups (*p* = 0.62).

### Sub‐analysis classified by the type of disease, age, and sex

We performed a subgroup analysis based on the type of disease (144 cases of MPA, 34 cases of GPA), age (84 patients aged 75 years and older, 94 patients aged less than 75 years), and sex (78 males, 100 females). The Kaplan–Meier curves of survival rate, relapse rate, and ESRD progression rate, and GC‐remission rates for the RTX and IVCY groups after IPTW analysis in each subgroup were shown in Fig. S3–S8. In the patient group aged 75 years and older, the 10‐year survival rate was significantly higher in the RTX group than in the IVCY group (Fig. S5a). In the patient groups with MPA, GPA, those aged less than 75 years, and females, the GC‐remission rate at 6 months was significantly higher in the RTX group than in the IVCY group (Figs. S3b, S4b, S6b, S8b). In the patient groups with MPA, GPA, and males, 10‐year relapse rates tended to be lower in the RTX group than in the IVCY group, but no significant difference was observed (Figs. 3c, S4c, S7c).

## Discussion

In this multicenter cohort study, the 10‐year survival rate was significantly higher in the RTX group than in the IVCY group. Additionally, the GC‐remission rate at 6 months was superior in the RTX group than in the IVCY group. The relapse and ESRD progression rates were comparable between the two groups. The proportion of severe infection‐related deaths was significantly lower in the RTX group than in the IVCY group.

Several reports have examined the differences in survival rates between the RTX and IVCY groups, but the superiority of RTX over IVCY in terms of survival has not yet been demonstrated. Ishikawa et al. reported that the 30‐day and 60‐day survival rates of Japanese patients with AAV were comparable between the RTX and IVCY groups [8]. Second, Casal Moura et al. showed that in patients with AAV and severe renal involvement, there was no difference in survival rates at 24 months between the IVCY and RTX groups [9]. However, these studies were retrospective with short follow‐up periods and could not compare long‐term mortality rates. In this study, we adjusted for patient background using the IPTW method and compared the long‐term survival rates of Japanese patients with AAV. To the best of our knowledge, this is the first report to demonstrate a significantly higher survival rate in the RTX group than in the IVCY group in severe AAV.

Previous studies have highlighted the superiority of RTX over IVCY in achieving steroid remission in patients with AAV. Puéchal et al. reported that the GC‐remission rate in patients with GPA at 6 months was significantly higher in the RTX group [11]. Additionally, Wallace et al. reported that patients treated with RTX were more likely to discontinue or achieve a lower dose of PDN within 6 months of treatment initiation [10]. Unizony et al. reported that the percentage of prednisone taper completion at 6 months was significantly higher in the RTX group than in the cyclophosphamide/AZP group among 131 patients with PR3‐AAV‐positive AAV [12]. The results of these studies were consistent with those of our study, showing that the RTX group is more likely to achieve GC remission at 6 months than the IVCY group.

One possible reason for the lower mortality in the RTX group may be its ability to taper PDN earlier at 6 months without disease worsening compared to the IVCY group. Previous reports have shown that a higher cumulative steroid dose is associated with an increased risk of severe infections [32]. Additionally, Xu et al. reported that respiratory infections during the 3 months after treatment are associated with increased mortality in patients with AAV [33]. These findings were consistent with our results, showing that infections were the leading cause of death in AAV. In our study, early tapering was possible in the RTX group at 3 months after treatment (Fig. 5). Early tapering of PDN may have contributed to the lower mortality rates due to severe infections, particularly respiratory infections, in the RTX group.

Several studies have examined the differences in relapse rates between the RTX and IVCY groups. The RITUXVAS trial showed that relapse rates did not differ between the two groups at 24 months [34]. In addition, Geetha et al. reported that in patients with AAV with renal involvement, the relapse rate at 18 months was 37% in the RTX group and 26% in the CYC group, with no significant difference between the two groups [13]. These findings are consistent with those of our study, showing that relapse rates are comparable between the two groups.

Regarding the ESRD progression rate, varying results in terms of RTX efficacy have been observed depending on the follow‐up period. In the short term, poor renal outcomes were more frequently observed in the RTX group than in the IVCY group [8]. In contrast, Wallace et al. reported that the 5‐year progression rate to renal failure was similar between the two groups [10]. In this study, ESRD progression rates were comparable between the two groups. The differences in these results may be influenced by factors such as racial differences, patient backgrounds, and treatment strategies. Further research is needed to determine whether ESRD progression and relapse rates are comparable between two groups.

Our study has several strengths. The REVEAL cohort study collected the clinical characteristics and several outcomes of 555 patients from multicenter institutes, enabling the analysis of representative real‐world AAV data. In addition, we clarified the differences in prognosis between the RTX and IVCY groups by adjusting for confounding factors using IPTW. Although our study has several strengths, it also has some limitations. First, the patients with AAV in our study were MPA‐dominant, and it is unclear whether the same results would apply to patients in Western countries, where GPA is more common [35]. Second, all participating centers in the REVEAL cohort were tertiary referral hospitals, which may have introduced a selection bias. Third, variations in treatment protocols across the multicenter cohort, such as the speed of PDN tapering and the rate of immunosuppressive agent use as maintenance therapy, may have influenced the outcomes. Fourth, the number of RTX group was smaller compared to that of IVCY group in this cohort. This is because in Japan, RTX was approved in AAV for public insurance coverage in 2013, which is later than the approval of IVCY [36]. Advances in supportive care, infection prevention, and diagnostic practices could be potential confounding factors for prognosis of AAV, given that RTX has been used more frequently in recent cases compared to IVCY. A future prospective study is needed to investigate the prognosis of patients with AAV in both the RTX and IVCY groups. However, despite these limitations, this study is significant because the use of the largest multicenter cohort database of AAV enhances the generalizability of our findings.

In conclusion, we revealed that RTX remission induction therapy has superior effectiveness in reducing mortality and achieving GC remission compared to IVCY treatment. These findings reveal the efficacy and safety of RTX remission induction therapy in real‐world Japanese clinical practice.

## Author contributions

Shogo Matsuda and Takuya Kotani designed the study. Daisuke Nishioka, Ayana Okazaki, Yuichi Masuda, TT, MS, Tsuneyasu Yoshida, Ryosuke Hiwa, MS, Ryu Watanabe, MH, Naoko Ito, Yohei Fujiki, Hirofumi Miyake, Wataru Yamamoto, MH carried out the acquisition of data. Shogo Matsuda and Daisuke Nishioka analyzed the data. Shogo Matsuda wrote the manuscript. Shogo Matsuda, Takuya Kotani, TT revised the manuscript.

## Conflict of interest statement

Shogo Matsuda has received research grant from Japan Intractable Diseases Research Foundation and Promotion and Mutual Aid Corporation for Private Schools of Japan. Takuya Kotani received speaker fees from AbbVie, Asahi Kasei, Boehringer Ingelheim, Chugai, Eisai, Eli Lilly, GlaxoSmithKline K.K., Kissei, and Taisho. Ryosuke Hiwa received research grants and/or speaker fees from AbbVie, Asahi Kasei, Bristol‐Myers Squibb, Daiichi Sankyo, Eisai, Eli Lilly, GlaxoSmithKline K.K., Kissei, Pfizer, Tanabe Mitsubishi, and UCB Japan. Ryu Watanabe received research grant from AbbVie and speaker fees from AbbVie, Asahi Kasei, Chugai, Eli Lilly, and GlaxoSmithKline K.K. Hirofumi Miyake received speaker fees from AbbVie, Asahi Kasei, AstraZeneca, Boehringer Ingelheim, Bristol‐Myers Squibb, Chugai, Eisai, Eli Lilly, GlaxoSmithKline K.K., Sanofi, and Tanabe Mitsubishi. Motomu Hashimoto received research grants and/or speaker fees from AbbVie, Asahi Kasei, Astellas, Bristol‐Myers Squibb, Chugai, Daiichi Sankyo, EA Pharma, Eisai, Eli Lilly, Novartis Pharma, Taisho Toyama, Tanabe Mitsubishi, Towa Pharma, and UCB Japan. Tohru Takeuchi received research grants and/or speaker fees from AbbVie, Asahi Kasei, Astellas, Bristol‐Myers Squibb, Chugai, Eisai, Eli Lilly, Janssen Pharma, Nihon Shinyaku, Mitsubishi‐Tanabe, Takeda, and Pfizer. Other authors (Daisuke Nishioka, Ayana Okazaki, Yuichi Masuda, Tomoki Taniguchi, Mikihito Shoji, Tsuneyasu Yoshida, Mayu Shiomi, Muneyuki Hatta, Naoko Ito, Yohei Fujiki, Wataru Yamamoto) do not have any conflict of interest.

## Supporting information




**Figure S1**: The details of immunosuppressive therapy as maintenance therapy for the IVCY and RTX groups at a time of relapse after IPTW analysis: (a) IVCY group and (b) RTX group. The proportions of immunosuppressive therapy were shown for each group. IVCY: intravenous cyclophosphamide, RTX: rituximab, IPTW: inverse probability of treatment weighting. AZA: azathioprine; MTX: methotrexate; MZB: mizoribine; CyA: cyclosporine.

**Figure S2**: The details of infections that caused hospitalization for the RTX and IVCY groups after IPTW analysis: (a) RTX group and (b) IVCY group. The percentages of infectious diseases were shown for RTX group and IVCY group. RTX: rituximab, IVCY: intravenous cyclophosphamide, IPTW: inverse probability of treatment weighting, MAC: Mycobacterium avium complex.

**Figure S3**: Over survival rates, GC‐remission rates, relapse rates, and ESRD rates in the IVCY and RTX groups after IPTW analysis of patients with MPA: (a) over survival rates, (b) GC‐remission ratios at month 6, (c) relapse‐free survival rates, (d) ESRD‐free survival rates in the IVCY and RTX groups after IPTW analysis. GC: glucocorticoid, ESRD: end‐stage renal disease, IVCY: intravenous cyclophosphamide, RTX: rituximab, IPTW: inverse probability of treatment weighting, MPA: microscopic polyangiitis.

**Figure S4**: Over survival rates, GC‐remission rates, relapse rates, and ESRD rates in the IVCY and RTX groups after IPTW analysis of patients with GPA: (a) over survival rates, (b) GC‐remission ratios at month 6, (c) relapse‐free survival rates, (d) ESRD‐free survival rates in the IVCY and RTX groups after IPTW analysis. GC: glucocorticoid, ESRD: end‐stage renal disease, IVCY: intravenous cyclophosphamide, RTX: rituximab, IPTW: inverse probability of treatment weighting, GPA: granulomatosis with polyangiitis.

**Figure S5**: Over survival rates, GC‐remission rates, relapse rates, and ESRD rates in the IVCY and RTX groups after IPTW analysis of patients with aged 75 years and older: (a) over survival rates, (b) GC‐remission ratios at month 6, (c) relapse‐free survival rates, (d) ESRD‐free survival rates in the IVCY and RTX groups after IPTW analysis. GC: glucocorticoid, ESRD: end‐stage renal disease, IVCY: intravenous cyclophosphamide, RTX: rituximab, IPTW: inverse probability of treatment weighting.

**Figure S6**: Over survival rates, GC‐remission rates, relapse rates, and ESRD rates in the IVCY and RTX groups after IPTW analysis of patients with aged less than 75 years: (a) over survival rates, (b) GC‐remission ratios at month 6, (c) relapse‐free survival rates, (d) ESRD‐free survival rates in the IVCY and RTX groups after IPTW analysis. GC: glucocorticoid, ESRD: end‐stage renal disease, IVCY: intravenous cyclophosphamide, RTX: rituximab, IPTW: inverse probability of treatment weighting.

**Figure S7**: Over survival rates, GC‐remission rates, relapse rates, and ESRD rates in the IVCY and RTX groups after IPTW analysis of male patients: (a) over survival rates, (b) GC‐remission ratios at month 6, (c) relapse‐free survival rates, (d) ESRD‐free survival rates in the IVCY and RTX groups after IPTW analysis. GC: glucocorticoid, ESRD: end‐stage renal disease, IVCY: intravenous cyclophosphamide, RTX: rituximab, IPTW: inverse probability of treatment weighting.

**Figure S8**: Over survival rates, GC‐remission rates, relapse rates, and ESRD rates in the IVCY and RTX groups after IPTW analysis of female patients: (a) over survival rates, (b) GC‐remission ratios at month 6, (c) relapse‐free survival rates, (c) ESRD‐free survival rates in the IVCY and RTX groups after IPTW analysis. GC: glucocorticoid, ESRD: end‐stage renal disease, IVCY: intravenous cyclophosphamide, RTX: rituximab, IPTW: inverse probability of treatment weighting.

**Table S1**: Baseline clinical characteristics and disease severity classification in patients with AAV.

**Table S2**: Context of immunosuppressants in patients with AAV.

**Table S3**: The detailed cause of death in IVCY group and RTX group after IPTW.

**STROBE Statement**—Checklist of items that should be included in reports of ***cohort studies***.

## Data Availability

The data that support the findings of this study are available on request from the corresponding author. The data are not publicly accessible due to privacy or ethical restrictions.
