# Supplementary figures and images for: Long‐term efficacy of rituximab versus intravenous cyclophosphamide for severe ANCA‐associated vasculitis in multicenter REVEAL cohort study
Source: J Intern Med. 2025 Sep 22;298(5):504–15. doi: 10.1111/joim.70024 (PMC12522534; doi:10.1111/joim.70024)

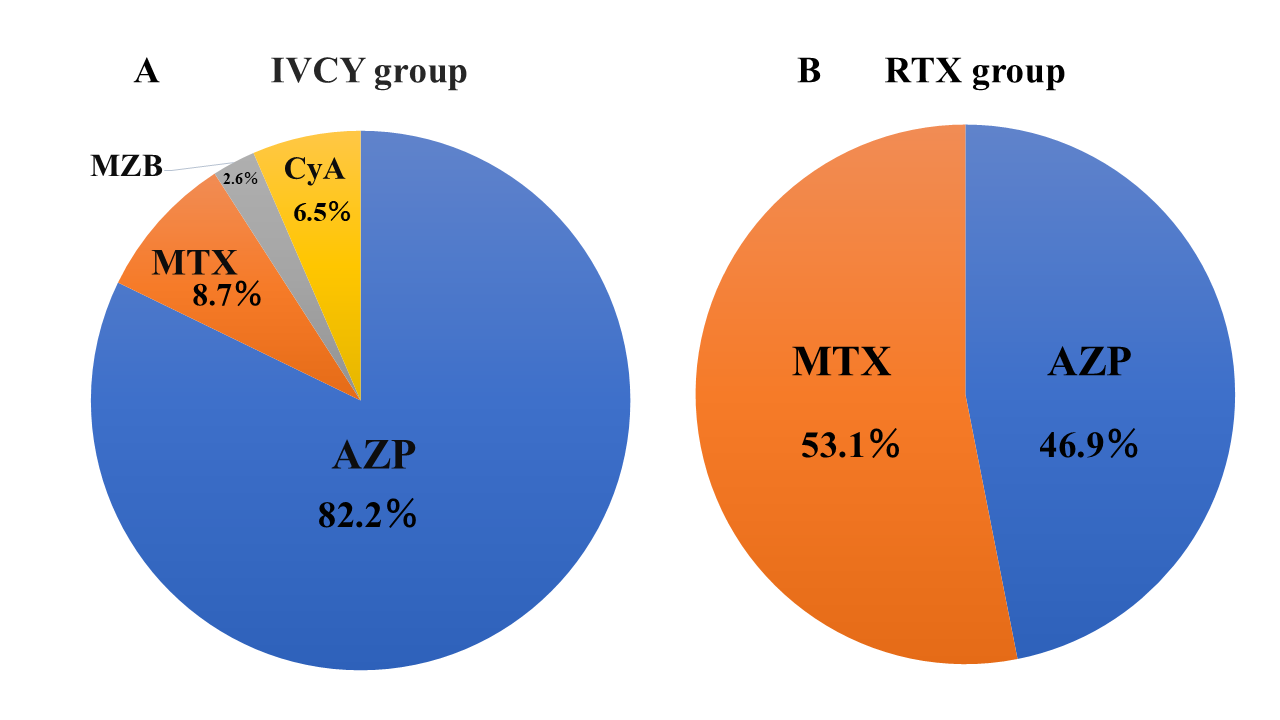

Supplement: Supplementary file 1 — Figure S1: The details of immunosuppressive therapy as maintenance therapy for the IVCY and RTX groups at a time of relapse after IPTW analysis: (a) IVCY group and (b) RTX group. The proportions of immunosuppressive therapy were shown for each group. IVCY: intravenous cyclophosphamide, RTX: rituximab, IPTW: inverse probability of treatment weighting. AZA: azathioprine; MTX: methotrexate; MZB: mizoribine; CyA: cyclosporine. [file JOIM-298-504-s012.tif]

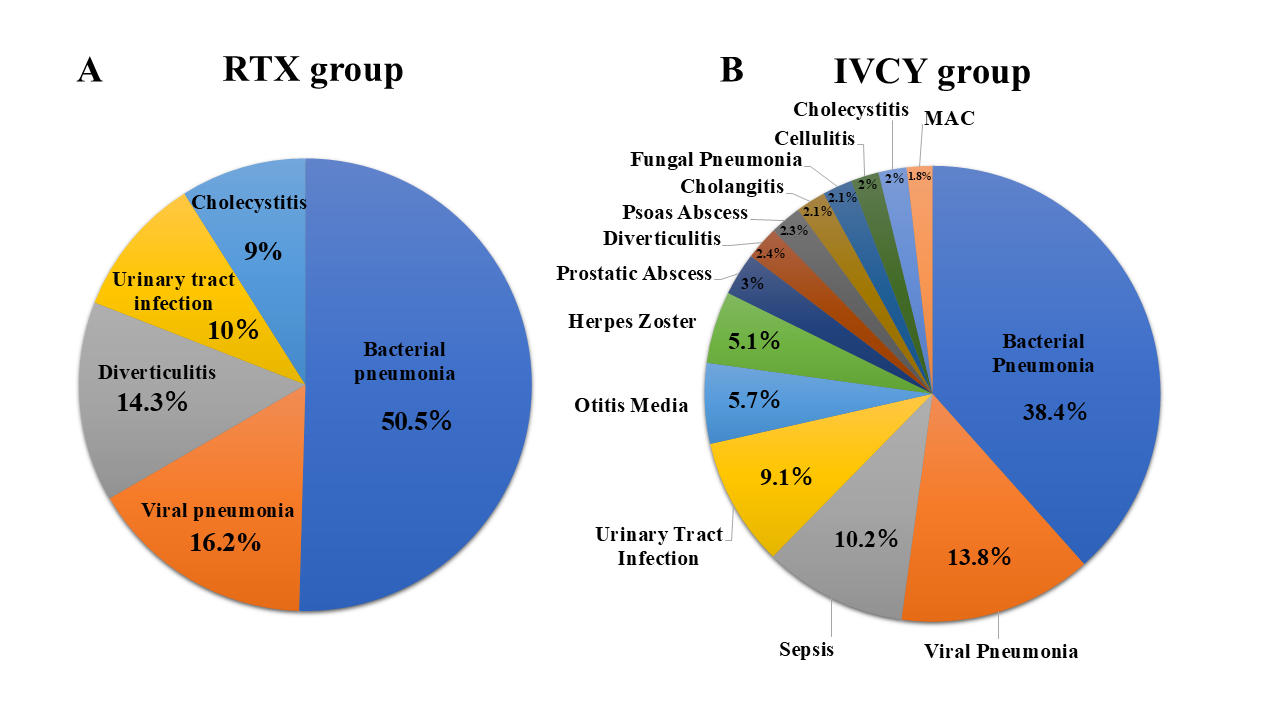

Supplement: Supplementary file 2 — Figure S2: The details of infections that caused hospitalization for the RTX and IVCY groups after IPTW analysis: (a) RTX group and (b) IVCY group. The percentages of infectious diseases were shown for RTX group and IVCY group. RTX: rituximab, IVCY: intravenous cyclophosphamide, IPTW: inverse probability of treatment weighting, MAC: Mycobacterium avium complex. [file JOIM-298-504-s003.tif]

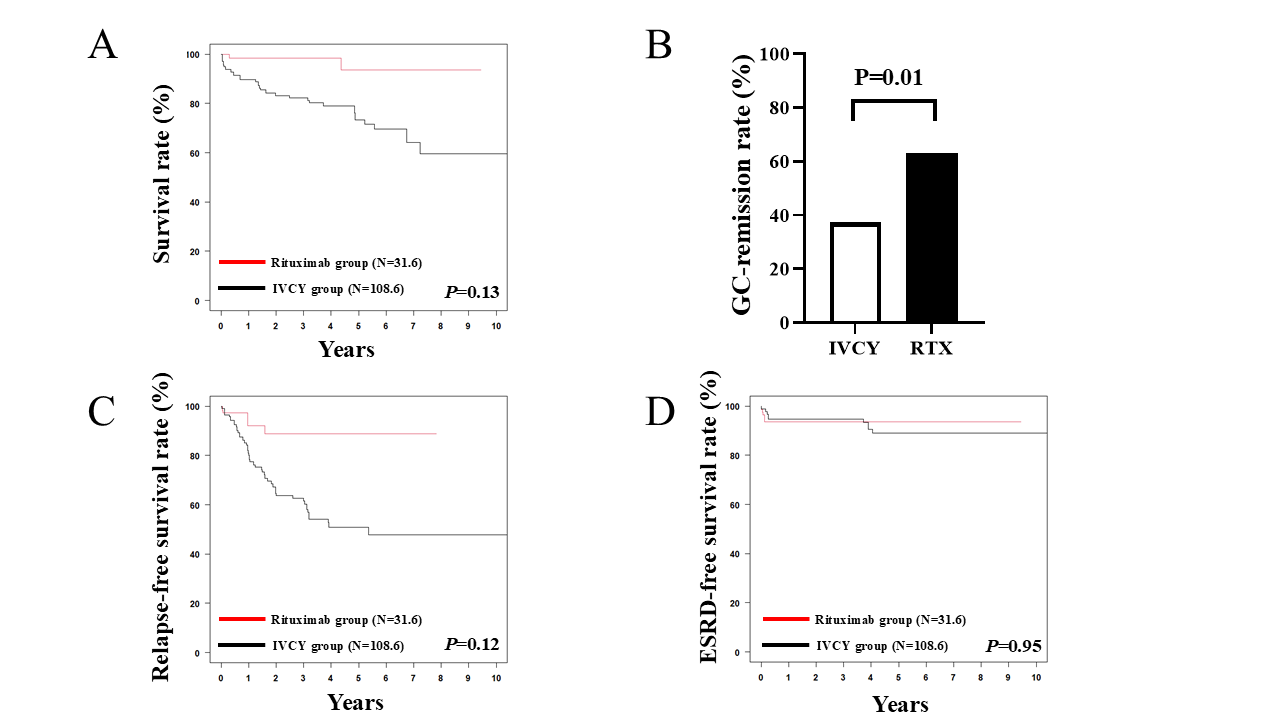

Supplement: Supplementary file 3 — Figure S3: Over survival rates, GC‐remission rates, relapse rates, and ESRD rates in the IVCY and RTX groups after IPTW analysis of patients with MPA: (a) over survival rates, (b) GC‐remission ratios at month 6, (c) relapse‐free survival rates, (d) ESRD‐free survival rates in the IVCY and RTX groups after IPTW analysis. GC: glucocorticoid, ESRD: end‐stage renal disease, IVCY: intravenous cyclophosphamide, RTX: rituximab, IPTW: inverse probability of treatment weighting, MPA: microscopic polyangiitis. [file JOIM-298-504-s001.tif]

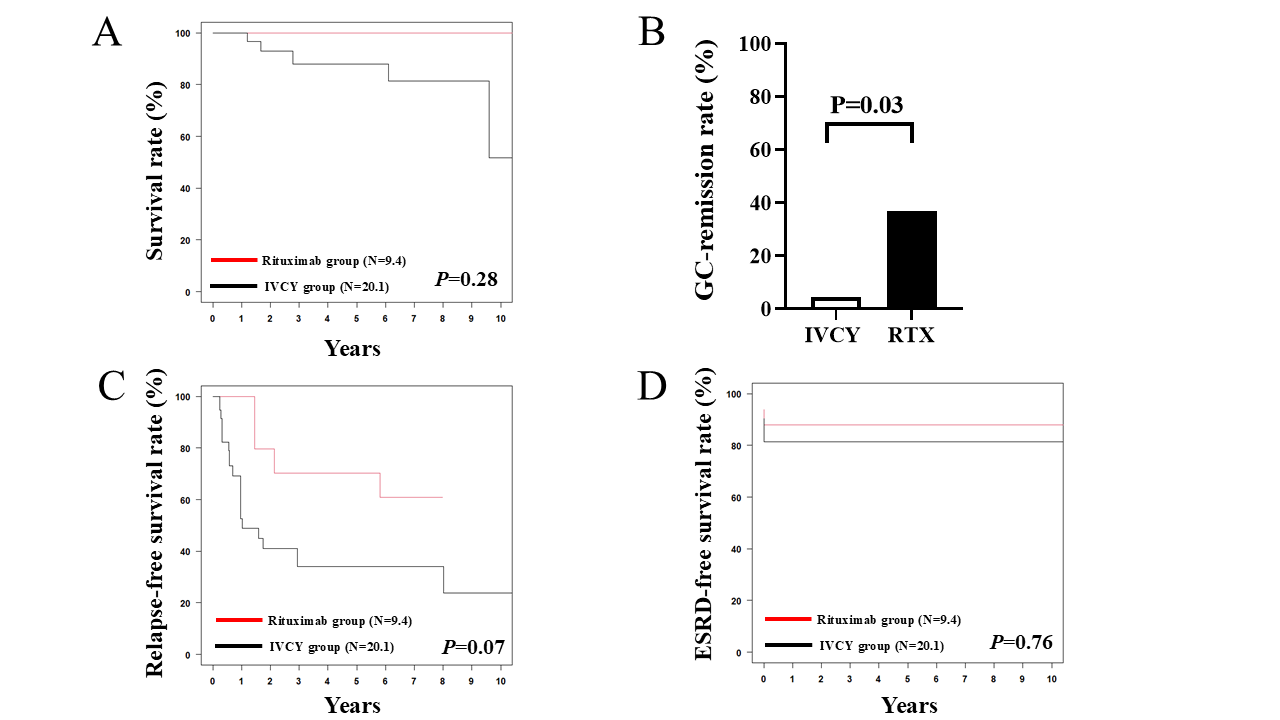

Supplement: Supplementary file 4 — Figure S4: Over survival rates, GC‐remission rates, relapse rates, and ESRD rates in the IVCY and RTX groups after IPTW analysis of patients with GPA: (a) over survival rates, (b) GC‐remission ratios at month 6, (c) relapse‐free survival rates, (d) ESRD‐free survival rates in the IVCY and RTX groups after IPTW analysis. GC: glucocorticoid, ESRD: end‐stage renal disease, IVCY: intravenous cyclophosphamide, RTX: rituximab, IPTW: inverse probability of treatment weighting, GPA: granulomatosis with polyangiitis. [file JOIM-298-504-s002.tif]

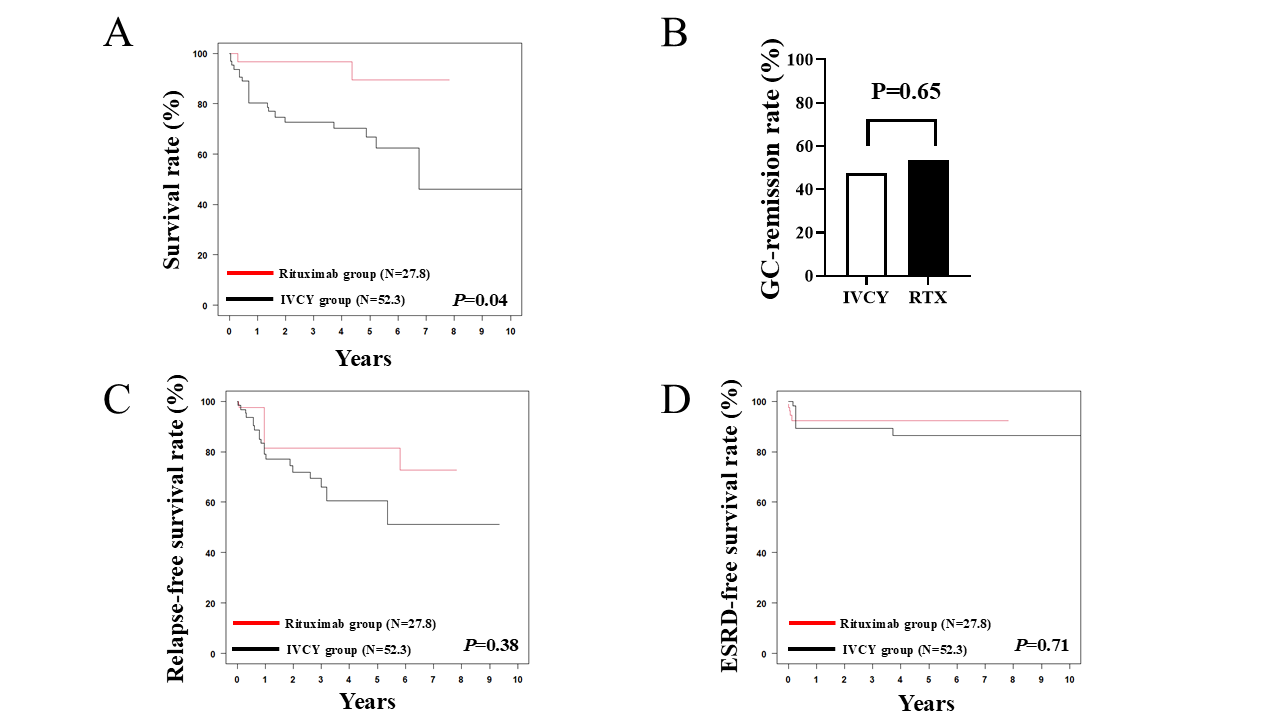

Supplement: Supplementary file 5 — Figure S5: Over survival rates, GC‐remission rates, relapse rates, and ESRD rates in the IVCY and RTX groups after IPTW analysis of patients with aged 75 years and older: (a) over survival rates, (b) GC‐remission ratios at month 6, (c) relapse‐free survival rates, (d) ESRD‐free survival rates in the IVCY and RTX groups after IPTW analysis. GC: glucocorticoid, ESRD: end‐stage renal disease, IVCY: intravenous cyclophosphamide, RTX: rituximab, IPTW: inverse probability of treatment weighting. [file JOIM-298-504-s006.tif]

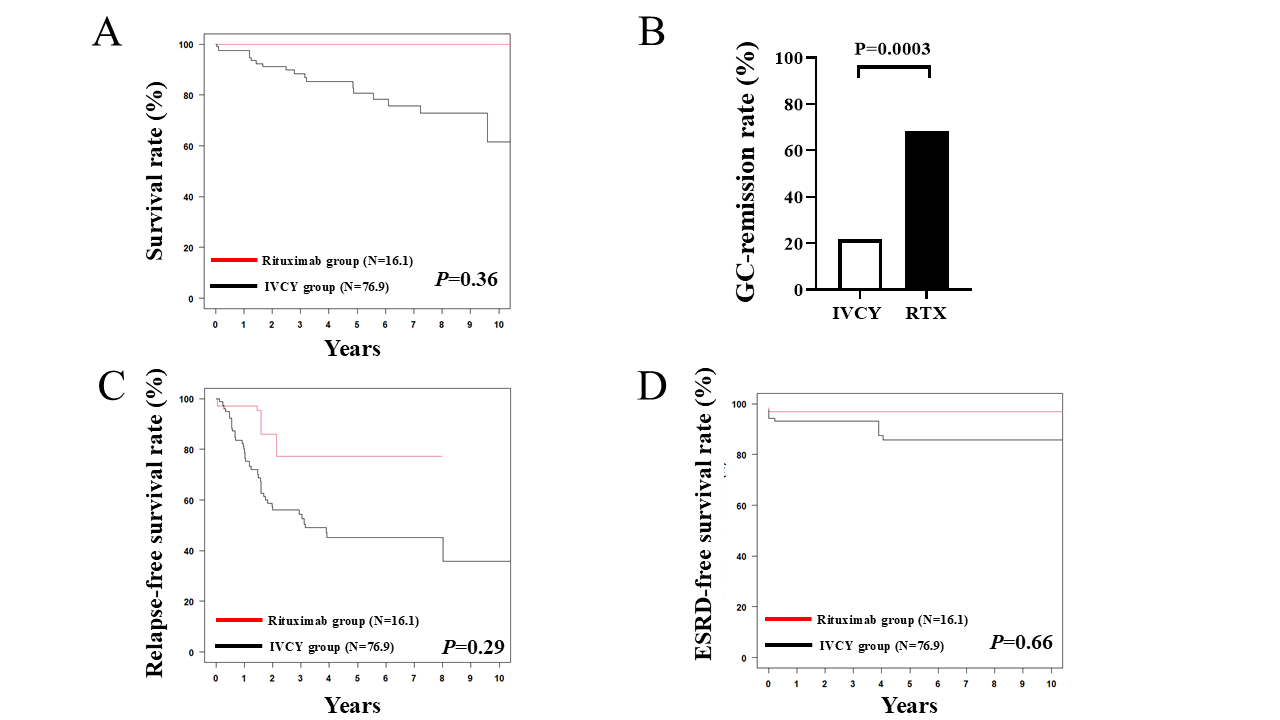

Supplement: Supplementary file 6 — Figure S6: Over survival rates, GC‐remission rates, relapse rates, and ESRD rates in the IVCY and RTX groups after IPTW analysis of patients with aged less than 75 years: (a) over survival rates, (b) GC‐remission ratios at month 6, (c) relapse‐free survival rates, (d) ESRD‐free survival rates in the IVCY and RTX groups after IPTW analysis. GC: glucocorticoid, ESRD: end‐stage renal disease, IVCY: intravenous cyclophosphamide, RTX: rituximab, IPTW: inverse probability of treatment weighting. [file JOIM-298-504-s007.tif]

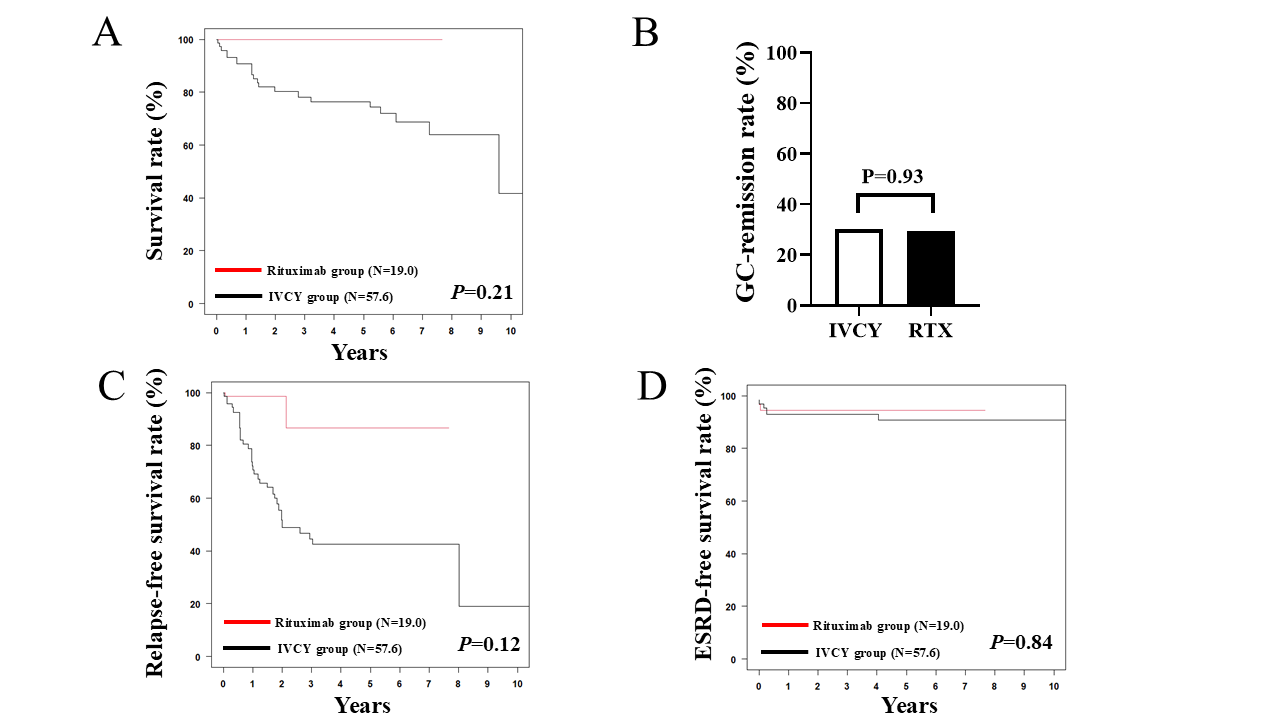

Supplement: Supplementary file 7 — Figure S7: Over survival rates, GC‐remission rates, relapse rates, and ESRD rates in the IVCY and RTX groups after IPTW analysis of male patients: (a) over survival rates, (b) GC‐remission ratios at month 6, (c) relapse‐free survival rates, (d) ESRD‐free survival rates in the IVCY and RTX groups after IPTW analysis. GC: glucocorticoid, ESRD: end‐stage renal disease, IVCY: intravenous cyclophosphamide, RTX: rituximab, IPTW: inverse probability of treatment weighting. [file JOIM-298-504-s009.tif]

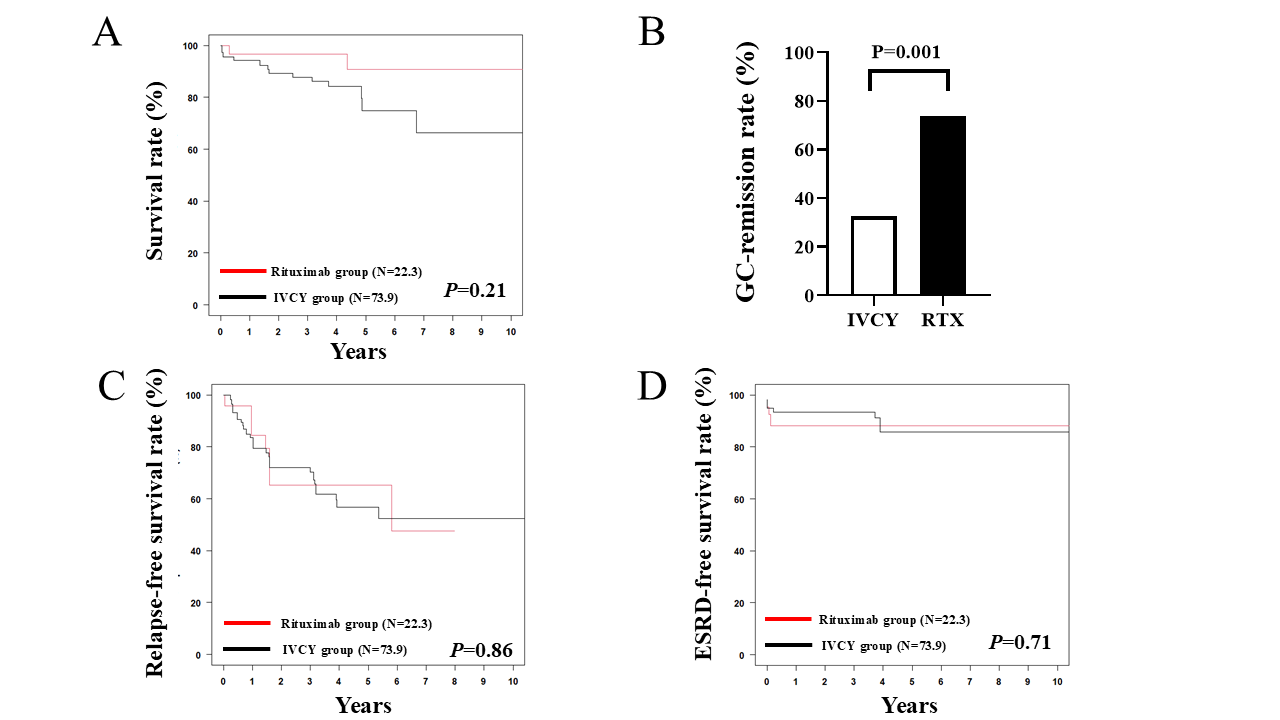

Supplement: Supplementary file 8 — Figure S8: Over survival rates, GC‐remission rates, relapse rates, and ESRD rates in the IVCY and RTX groups after IPTW analysis of female patients: (a) over survival rates, (b) GC‐remission ratios at month 6, (c) relapse‐free survival rates, (c) ESRD‐free survival rates in the IVCY and RTX groups after IPTW analysis. GC: glucocorticoid, ESRD: end‐stage renal disease, IVCY: intravenous cyclophosphamide, RTX: rituximab, IPTW: inverse probability of treatment weighting. [file JOIM-298-504-s008.tif]
