# Supplementary material for: Long‐term efficacy of rituximab versus intravenous cyclophosphamide for severe ANCA‐associated vasculitis in multicenter REVEAL cohort study
Source: J Intern Med. 2025 Sep 22;298(5):504–15. doi: 10.1111/joim.70024 (PMC12522534; doi:10.1111/joim.70024)
Supplement: Supplementary file 9 — Table S1: Baseline clinical characteristics and disease severity classification in patients with AAV. [file JOIM-298-504-s010.docx]

**Table S1. Baseline clinical characteristics, disease severity classification in patients with AAV**

| Variables | N=178 |
| --- | --- |
| Age, years | 74（67-79） |
| Female, n (%) | 100（56.2） |
| MPA, n (%) | 144 (80.9) |
| GPA, n (%) | 34 (19.1) |
| MPO-ANCA positivity, n (%) | 155 (87.0) |
| PR3-ANCA positivity, n (%) | 20 (11.2) |
| ILD, n (%) | 75 (42.1) |
| BVAS at onset | 17 (12-20) |
| **Laboratory findings** |  |
| WBC, /mm3 | 11325 (8083-14335) |
| CRP, mg/dl | 8.6 (2.4-13.8) |
| Cr, mg/dl | 1.13 (0.71-2.08) |
| **Organ involvements** |  |
| General, n (%) | 120 (67.4) |
| Cutaneous, n (%) | 20 (11.2) |
| Mucous membranes/eyes, n (%) | 20 (11.2) |
| ENT, n (%) | 46 (25.8) |
| Chest, n (%) | 93 (52.2) |
| Cardiovascular, n (%) | 7 (3.9) |
| Abdominal, n (%) | 2 (1.1) |
| Renal, n (%) | 134 (75.3) |
| Nervous system, n (%) | 71 (39.9) |
| **EUVAS-defined disease activity** |  |
| Localized, n (%) | 3 (1.7) |
| Early systemic, n (%) | 35 (19.7) |
| Systemic, n (%) | 111 (62.4) |
| Severe, n (%) | 29 (16.3) |
| **Remission induction therapy** |  |
| Initial PDN dose, mg/kg | 1.0 (0.96-1.0) |
| MPDN pulse, n (%) | 70 (39.3) |
| IVCY, n (%) | 133 (74.7) |
| RTX, n (%) | 45 (25.3) |

The laboratory markers are presented as the median (interquartile range). AAV: Antineutrophil cytoplasmic antibody-associated vasculitis; MPA: microscopic polyangiitis; GPA: granulomatosis with polyangiitis; MPO-ANCA: myeloperoxidase-anti-neutrophil cytoplasmic autoantibody; PR3-ANCA: proteinase 3-anti-neutrophil cytoplasmic antibody; ILD: interstitial lung disease; BVAS: Birmingham Vasculitis Activity Score; WBC: white blood cell; CRP: C-reactive protein; Cr: creatinine; ENT: ear, nose and throat; EUVAS: European Vasculitis Study Group; PDN: prednisolone; MPDN:methylprednisolone; IVCY: intravenous cyclophosphamide; RTX: rituximab.
