# Supplementary material for: Long‐term efficacy of rituximab versus intravenous cyclophosphamide for severe ANCA‐associated vasculitis in multicenter REVEAL cohort study
Source: J Intern Med. 2025 Sep 22;298(5):504–15. doi: 10.1111/joim.70024 (PMC12522534; doi:10.1111/joim.70024)
Supplement: Supplementary file 10 — Table S2: Context of immunosuppressants in patients with AAV. [file JOIM-298-504-s005.docx]

**Table S2. Context of Immunosuppressants in patients with AAV**

| **Immunosuppressants** | N=178 |
| --- | --- |
| **Maintenance therapy** |  |
| AZA, n (%) | 108 (60.7) |
| MTX, n (%) | 10 (5.6) |
| MMF, n (%) | 10 (5.6) |
| TAC, n (%) | 7 (3.9) |
| MZB, n (%) | 3 (1.7) |
| CyA, n (%) | 4 (2.2) |
| RTX, n (%) | 20 (11.2) |

Categorical variables are presented as number (%). AAV: Antineutrophil cytoplasmic antibody-associated vasculitis; AZA: azathioprine; MTX: Methotrexate; MMF: mycophenolate mofetil; TAC: tacrolimus; MZB: mizoribine; CyA: cyclosporine; RTX: rituximab.
