# Supplementary material for: Long‐term efficacy of rituximab versus intravenous cyclophosphamide for severe ANCA‐associated vasculitis in multicenter REVEAL cohort study
Source: J Intern Med. 2025 Sep 22;298(5):504–15. doi: 10.1111/joim.70024 (PMC12522534; doi:10.1111/joim.70024)
Supplement: Supplementary file 11 — Table S3: The detailed cause of death in IVCY group and RTX group after IPTW. [file JOIM-298-504-s011.docx]

**Table S3. The detailed cause of death in IVCY group and RTX group after IPTW**

| **The causes of death** | **IVCY group (N=133.6)** | **RTX group (N=42.2)** |
| --- | --- | --- |
| **AAV exacerbation n(%)** | **7.7 (5.8%)** | **0.3 (0.7%)** |
| **Infection, n(%)** | **20.3 (15.2%)** | **0 (0%)** |
| **Others, n(%)** | **13.7 (10.2%)** | **0.65 (1.5%)** |

IVCY: intravenous cyclophosphamide; RTX: rituximab; IPTW: Inverse Probability of Treatment Weighting; AAV: Antineutrophil cytoplasmic antibody-associated vasculitis.
